# *Borrelia theileri* infection in beef and dairy cattle in Israel: molecular detection and co-infection with bovine piroplasmids

**DOI:** 10.1186/s13071-026-07527-6

**Published:** 2026-06-23

**Authors:** Dor Shwartz, Harold Salant, Yaarit Nachum-Biala, Netta Simon, Monica Leszkowicz -Mazuz, Daniel Yasur-Landau, Dan Gleser, Eyal Klement, Nir Rudoler, Elad Eliahoo, Gad Baneth

**Affiliations:** 1 Koret School of Veterinary Medicine, Hebrew University of Jerusalem, P.O. Box 12, 7610001 Rehovot, Israel; 2 Division of Parasitology, Kimron Veterinary Institute, 5020001 Beit Dagan, Israel; 3 Department of Virology, Kimron Veterinary Institute, 5025001 Beit Dagan, Israel

**Keywords:** Tick-borne relapsing fever, *Borrelia theileri*, Bovine borreliosis, Bovine piroplasmosis

## Abstract

**Background:**

Tick-borne relapsing fever is an infectious disease caused by spirochetes of the genus *Borrelia,* leading to fever and spirochetemia in humans and animals. *Borrelia theileri* is the agent of bovine relapsing fever, which is transmitted by ixodid ticks of the genus *Rhipicephalus* and has been reported to cause mild disease in cattle. It has been infrequently reported in several countries over a broad geographic area; however, it has not been reported in Israel previously.

**Methods:**

Blood samples were collected from beef and dairy cattle in 23 locations in Israel. Real-time and conventional PCR were used to detect *Borrelia* spp. and co-infection with bovine piroplasmids. PCR products were sequenced, and phylogenetic analysis of the *B. theileri* sequences was performed. Positive cattle were identified in a limited number of locations with no clear spatial clustering.

**Results:**

*Borrelia theileri* DNA was detected in 6 out of 439 (1.4%) blood samples. Of these, four were from beef and two from dairy cattle. No significant differences were found according to age (*P* = 0.642) and production type (*P* = 0.802) among the *B. theileri*-positive cattle. Phylogenetic analysis showed clustering of the cattle-derived isolate together with *B. theileri* amplified from ixodid ticks, supporting a potential transmission cycle involving cattle and *Rhipicephalus* spp. ticks. *Theileria annulata* and *Babesia bigemina* DNA was detected in 11 (4.1%) and one (0.4%) of 268 unvaccinated cattle, respectively. Co-infection with *Theileria annulata* was found in one beef and one dairy cattle.

**Conclusions:**

Tick-borne relapsing fever is present in the cattle population of Israel. Co-infection of *B. theileri* and bovine piroplasmids may drive clinical disease and financial losses in beef and dairy cattle. This is the first report of *B. theileri* infection in Israel. Future control programs should address the exposure of both beef and dairy cattle to ticks and tick-borne diseases.

**Graphical Abstract:**

**Figure:**
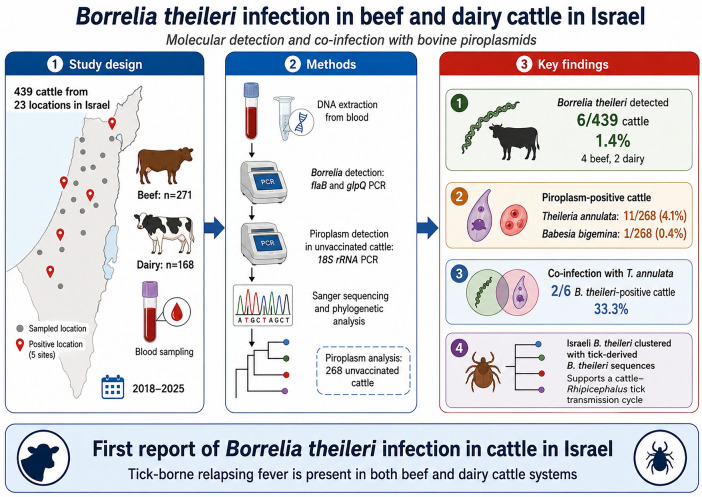

## Background

Tick-borne relapsing fever (TBRF) *Borrelia* spp. cause infectious disease affecting humans and animals [1]. Relapsing fever (RF) in humans is characterized by episodes of recurrent fever, lethargy, headache, vomiting, muscle pain, and joint pain, with up to 10% mortality in untreated patients [2]. While most TBRF *Borrelia* spp. are transmitted by argasid ticks of the genus *Ornithodoros*, a group of hard tick-borne RF species was described and includes *Borrelia miyamotoi,* transmitted by ticks of the genus *Ixodes*, *Borrelia theileri,* transmitted by *Rhipicephalus* spp., and *Borrelia lonestari* transmitted by *Amblyomma americanum* [3–6]. *Borrelia persica* transmitted by the argasid tick *Ornithodoros tholozani*, which affects people in the eastern Mediterranean basin and Asia, was considered the only causative agent of TBRF in Israel [7–9]. Previous studies described TBRF infection in animals in Israel including domestic dogs (*Canis lupus familiaris*) and cats (Felis catus), in which infection is associated with clinical disease, and wildlife animal species including several rodent species (*Microtus socialis, Psammomys obesus, Acomys cahirinus*), the rock hyrax (*Procavia capensis*), red fox (*Vulpes vulpes*), golden jackal (*Canis aureus*), European badger (*Meles meles*), and the striped hyena (*Hyaena hyaena*) [10–13].

*Borrelia theileri* was described in 1905 as a spiral bacterium infecting cattle and transmitted by ixodid ticks [14]. In contrast to other TBRF species, clinical, and epidemiological data regarding *B. theileri* infection is sparse and includes sporadic reports of molecular detection in domestic and wild mammalian species. These reports include cattle (*Bos taurus*) and impalas (*Aepyceros melampus*) in Zambia, sheep (*Ovis aries*), and goats (*Capra hircus*) in Egypt and Algeria, and racoon dogs (*Nyctereutes procyonoides*) in Korea [15–18]. Reports of molecular detection of *B. theileri* in dairy cows in Brazil were associated with a drop in milk production and reduction in feed consumption [19]. Other reports from beef cattle in Korea and impalas in Zambia, did not include observation of clinical signs [18, 20]. *Borrelia theileri* infection was detected in several *Rhipicephalus* tick species, including *R. annulatus*, *R. microplus,* *R.* *turanicus*, and *R. geigyi* in, Iran, Brazil, Pakistan, and Mali [21–24]. An unusual finding of *B. theileri* detection in the human head lice (*Pediculus humanus capitis*) was also reported in the Republic of Congo [25]. Co-infection of *B. theileri* with bovine piroplasmosis agents, including *Babesia bovis*,* B. bigemina*, and *Theileria annulata* was described in *R. annulatus* ticks in Egypt; however, data regarding co-infection of these pathogens in cattle are limited and have only been reported in a single case report describing *B. theileri* infection concurrent with *B. bovis* and *Theileria mutans* in an ox and a cow in Botswana [26, 39].

As global dairy and beef food markets were estimated at approximately 700 billion USD in 2024 (https://www.fao.org/faostat), under-recognized burdens to production are of great concern for this industry. The annual economic losses due to tick-borne diseases are estimated at 14–19 billion USD in developing countries [27]. Therefore, detection of previously unrecognized infectious agents in cattle is important and may contribute to reducing economic losses. The aim of this study was to survey beef and dairy cattle in Israel for infection with *Borrelia* spp. and to evaluate potential co-infection with agents of bovine piroplasmosis in infected animals.

## Methods

### Sample collection

Bovine whole blood samples used in the current study were collected between 2018 and 2025 from beef and dairy cattle in Israel as part of vector-borne disease monitoring surveys (bovine piroplasmosis, Crimean-Congo hemorrhagic fever virus, and bovine ephemeral virus). The sampled cattle were distributed across northern Mediterranean climate regions, central semi-arid zones, and southern arid/desert areas in Israel. These areas are characterized by a gradient of decreasing annual rainfall and increasing temperature from north to south. Samples were collected from clinically healthy cattle, and from herds where clinical evidence of bovine piroplasmosis was detected. All cattle were vaccinated for Foot and Mouth Disease virus and for bovine brucellosis, according to the Israeli Ministry of Agriculture mandatory vaccination program. Whole blood samples were collected in EDTA anticoagulated tubes. Recorded data obtained from the animals’ medical records included sex, age (calf, ≤ 1 year; heifer, 1–2 years; adult ≥ 2 years), production type (beef or dairy), and farm location. Data on tick infestation was not available for the animals at the time of sampling.

Blood samples were preserved at − 20 °C until DNA extraction. DNA was extracted from 200 µl blood using a commercial kit (DNeasy Blood & Tissue Kit, Qiagen, Germany) following the manufacturer’s protocol.

### Molecular identification and characterization of *Borrelia* and piroplasm spp.

Real-time and conventional polymerase chain reactions (PCR) were performed on DNA extracted from the blood samples by the amplification of two different genes of RF *Borreliae*. The fragments of the *Borrelia* flagellin (*flaB*) gene were amplified using real-time (346-bp) and conventional (750-bp) PCR as previously reported using primers Bfpbu/Bfpcr and BOR1/BOR2 [28, 29]. In addition, an approximately 280-bp fragment of the glycerophosphodiester phosphodiesterase (*glpQ*) gene, which is specific for RF *Borreliae*, was amplified using primers 510f and 770r [30]. The *flaB* and *glpQ* PCR protocols were carried out as previously described [13, 29]. DNA extracted from a *B. persica* culture grown in the authors’ laboratory was used as a positive control. In addition, to detect possible co-infection with bovine piroplasmosis agents, conventional PCR was performed to amplify segments of the *18S rRNA* gene from the bovine blood samples for the detection of *Babesia* spp. and *Theileria* spp. [31] (Table 1).

**Table 1:** Target genes and primers used for PCR to detect and characterize *Borrelia*, *Babesia*, and *Theileria* spp.

Pathogen	Target gene	Primers	Amplicon size (bp)	Primer sequence (5ʹ-3ʹ)	References
*Borrelia* spp.	*flaB*	Bfpbu	346	GCTGAAGAGCTTGGAATGCAACC	[28]
		Bfpcr		TGATCAGTTATCATTCTAATAGCA	
		BOR1	750	TAATACGTCAGCCATAAATGC	[29]
		BOR2		GCTCTTTGATCAGTTATCATTC	
	*GlpQ*	Glpq-510f	280	AAAACCCTTTTGGCATAAACAACA	[30]
		Glpq-770r		CCAGGGTCCAATTCCGTCAG	
*Babesia* spp. and *Theileria* spp.	*18S rRNA*	Piroplasmid-F	350	CCAGCAGCCGCGGTAATTC	[31]
		Piroplasmid-R		CTTTCGCAGTAGTTYGTCTTTAACAAATC	

Each *18S rRNA* PCR was performed in 25-μL reaction volume containing 4 μL of sample DNA, 1 μM of each primer, and 19 μL of distilled extra pure water [31]. DNA extracted from a *Babesia negevi* positive canine blood sample was used as a positive control. All PCR products were Sanger sequenced from both directions using the respective forward and reverse primers. Sequencing was performed by the BigDye Terminator cycle sequencing chemistry from Applied Biosystems ABI 3700 DNA Analyzer (ABI, Carlsbad, CA, USA) and the ABI Data collection and Sequence Analysis software at the Center for Genomic Technologies, Hebrew University of Jerusalem, Israel. DNA sequences were aligned using the Molecular Evolutionary Genetics Analysis software MEGA, version 11.013 [32]. Sequences were further compared to the GenBank database using the BLAST algorithm (National Center for Biotechnology Information, Bethesda, USA; http://blast.ncbi.nlm.nih.gov/Blast.cgi). Species-level identification was obtained according to the closest BLAST match when sequences showed identity higher than 99% to the first match GenBank sequences.

### Statistical analysis

Statistical analysis was performed using the SPSS version 28.0.1.1 (SPSS IBM, Armonk, NY, USA). Statistical significance was defined as *P* < 0.05. Categorical associations between infection status and age or production type were assessed by Pearson’s χ^2^ test.

Maps showing the collection sites were constructed using the ArcGIS Map 10.0 software (Esri, Redlands, CA, USA).

## Results

### Study population and molecular analyses

**Fig. 1:**
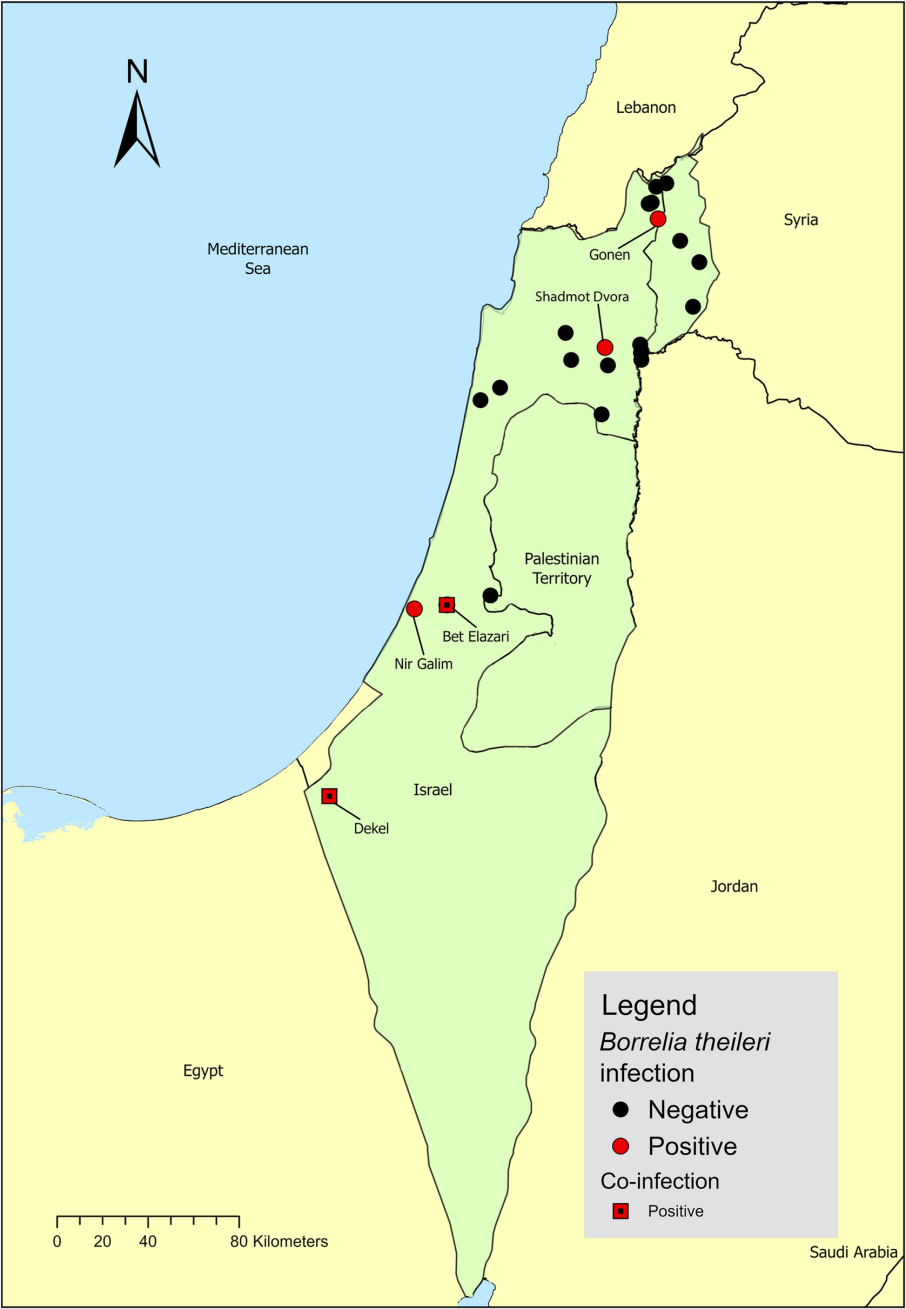
Geographical distribution of sampled cattle indicating infection rate for *B. theileri*. The black marks are representative of sampling locations without *B. theileri* infection presence, the red circle marks *B. theileri* positive sampling locations, and red squares with a centered black point represent locations where co-infection of *B. theileri* and *T. annulata* were detected

Samples were collected from a total of 437 cows and 2 bulls from 23 locations (Fig. 1). Of these, 271 (61.7%) were beef cattle and 168 (38.3%) were dairy cows. Samples were collected from a total of 50 calves (dairy only), 30 heifers (27 dairy and 3 beef), and 359 adults (268 beef and 91 dairy). Dairy cows were all kept under an intensive housing system, with no access to pasture, whereas beef cattle were raised under a pasture-based grazing system. A total of six cattle (1.4%; 95% CI 0.5–2.9%) were positive for *B. theileri* DNA by real-time PCR amplification and sequencing of the *flaB* and *glpQ* genes, and 12 cattle (4.47%; 95% CI 2.3–7.7%) were positive for bovine piroplasmid infection (Table 2). Of these, 11 cattle were found positive for *T. annulata,* and one positive for *B. bigemina*. Cattle which were previously vaccinated against bovine piroplasmosis (*n* = 171) were excluded from the piroplasmid infection analysis due to the possible detection of the DNA vaccine strains in their blood.

**Table 2:** Sampling locations, production type, and number of *B. theileri* and piroplasmid infected cattle per location. Cattle previously vaccinated against bovine piroplasmosis were excluded from the piroplasmid analysis

Farm location	Production type	Total samples	*Borrelia theileri* positive	Piroplasmid positive (unvaccinated)
Amikam	Beef	26	0	0
Binyamina	Beef	38	0	-
Dekel	Beef	3	2	1
Gazit	Beef	10	0	-
Gilboa	Beef	21	0	0
Gonen	Beef	19	1	-
Keshet	Beef	27	0	4
Kfar Szold	Beef	30	0	-
Nafah	Beef	24	0	-
Nov	Beef	11	0	0
Shadmot Dvora	Beef	4	1	1
Shaalvim	Beef	10	0	-
Snir	Beef	10	0	-
Tel Adashim	Beef	21	0	-
Zipori	Beef	17	0	-
Afikim	Dairy	30	0	0
Amir	Dairy	33	0	0
Ashdot Ya'akov	Dairy	31	0	1
Bet Elazari	Dairy	3	1	2
Dafna	Dairy	26	0	1
Dgania A	Dairy	12	0	0
Kfar Blum	Dairy	30	0	2
Nir Galim	Dairy	3	1	0
Total		439	6/439 (1.36%)	12/268 (4.47%)

- vaccinated herds

Co-infection of *B. theileri* with *T. annulata* was found in two out of six (33.3%) *B. theileri* positive cattle, including one dairy cow and one beef bull (Table 3). Among unvaccinated animals, piroplasm infection was associated significantly with *B. theileri* infection, where piroplasm positivity was detected in 33.3% (2/6) of *B. theileri*-positive animals and in 3.9% (10/255) of *B. theileri*-negative animals (Fisher’s exact test *P* = 0.029; OR = 11.4, 95% CI 0.9–87.3).

**Table 3:** Infection rates of *B. theileri, T. annulata,* and *B. bigemina* in beef and dairy cattle. Cattle previously vaccinated against bovine piroplasmosis were excluded from the piroplasmid analysis

Type	Total number of samples	Number of calves	Number of heifers	Number of adults	*B. theileri* positive	*T. annulata* positive (unvaccinated)	*B. bigemina* positive (unvaccinated)	*B. theileri* and *T. annulata* positive
Beef	271	0/271	3/271 (1.10%)	268/271 (98.9%)	4/271 (1.47%)	5/100 (5.00%)	1/100 (1.00%)	1/100 (1.00%)
Dairy	168	50/168 (29.70%)	27/168 (16.07%)	91/168 (54.16%)	2/168 (1.20%)	6/168 (3.57%)	0/168	1/168 (0.59%)
Total	439	50/439 (11.38%)	30/439 (6.83%)	359/439 (81.77%)	6/439 (1.36%)	11/268 (4.10%)	1/268 (0.37%)	2/268 (0.74%)

DNA sequence analysis of the *B. theileri* positive samples showed that they shared 100% identity between all sequences and 100% identity and coverage to *B. theileri* *flaB* GenBank accession number MG601737.1 amplified from a *R. microplus* tick in Brazil. One cow was also positive for the *glpQ* gene showing 100% cover and 99% identity to *B. theileri* GenBank accession number OR037294.1 amplified from a *R. annulatus* tick in Iran. Sequence analysis of the piroplasmid-positive samples showed 99.7% identity and 100% coverage to *T. annulata* GenBank accession number OR144141.1 in all *T. annulata*-positive samples, and 99.3% identity and 100% coverage to *B. bigemina* GenBank accession number EF458199.1 amplified from cattle in Turkey in the *B. bigemina* positive sample.

Geographical distribution of the cattle tested ranged from the village, Snir in northern Israel to the village, Dekel in the south. (Fig. 1). All *B. theileri* positive cattle were adults, of which four were beef cattle and two dairy cows. Positive cattle resided in five different locations across Israel from the villages of Shadmot Dvora and Gonen in the Northeastern part of Israel to Dekel in the Southwestern part of the country (Fig. 1).

All cattle positive for bovine piroplasmosis were adults, of which 1 male and 11 female. Furthermore, there were six beef and six dairy cows. No significant differences were found between age (χ^2^ = 1.35, *df* = 2, *P* = 0.642) and production type (χ^2^ = 0.063, *df* = 1, *P* = 0.802) among *B. theileri* positive cattle. Nevertheless, a higher infection rate was found in males (2/2, 100%) than in females (4/437, 0.9%; Fisher’s exact test *P* < 0.0001). Furthermore, no significant differences were found between production type (χ^2^ = 0.861, *df* = 1, *P* = 0.353) among piroplasmid positive cattle. However, the association between age group and sex with piroplasmid infection status were almost of statistical significance (χ^2^ = 5.268, *df* = 2, *P* = 0.059; Fisher’s exact test *P* = 0.088, respectively).

### Phylogenetic analyses

**Fig. 2:**
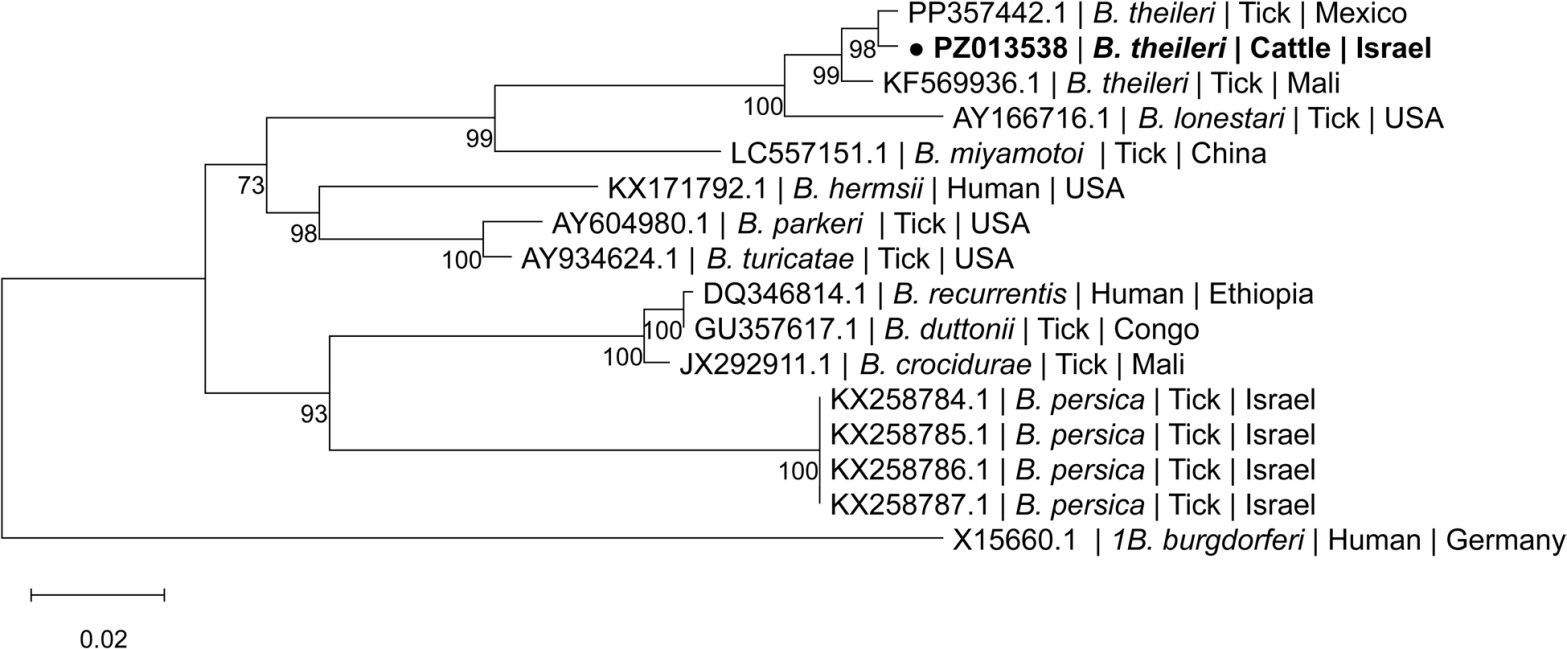
A maximum likelihood phylogram comparing 712-bp DNA sequences of the *flaB* gene from a positive Israeli cow to sequences from other *B. theileri* and *Borrelia* spp. The new sequence derived from this study is marked in bold letters and a black circle. The GenBank accession numbers, species of infected host, and country of origin are included for each sequence. The Tamura three-parameter model was used in the construction of this phylogram with bootstrap performed on 1000 replicates, and bootstrap values higher than 70 are indicated

Phylogenetic analysis based on a 712-bp segment of the *flaB* gene (Fig. 2) revealed that the *B. theileri* sequences from the current study clustered together with *B. theileri* previously amplified from ticks in Mexico and Mali (GenBank PP357442.1 and KF569936.1, respectively). All *B. theileri* sequences clustered separately from other Old World relapsing fever *Borrelia* spp. including *B. recurrentis*, *B. duttonii*, *B. crocidurae* and *B. persica*, and closer to TBRF species which are transmitted by ixodid ticks including *B. lonestari* and *B*. *miyamotoi*. North American RF species including *B. parkeri* and *B. turicatae* also clustered separately.

**Fig. 3:**
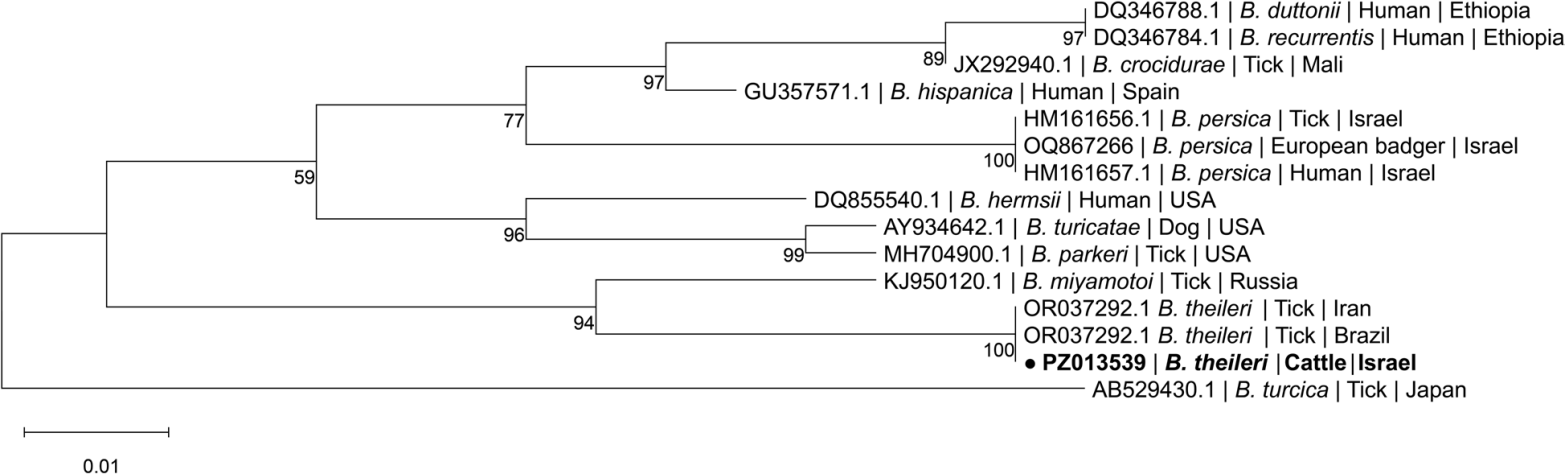
A maximum likelihood phylogram comparing 207-bp DNA fragment of the *glpQ* gene from a positive Israeli cow to other *B. theileri* and *Borrelia* spp. The new sequence derived from this study is written in bold letters and marked with a black circle. The GenBank accession numbers, species of infected host, and country of origin are included for each sequence. The Tamura three-parameter model was used in the construction of this phylogram with bootstrap performed on 1000 replicates, and values higher than 70 are indicated

Phylogenetic analysis based on a 207-bp segment of the *glpQ* gene (Fig. 3) also revealed that the sequence from the *B. theileri*-positive Israeli cattle clustered with other *B. theileri* sequences from ticks in Iran and Brazil (GenBank OR037292.1 and OR037292.1, respectively). As demonstrated in the *flaB* phylogram, *B. theileri glpQ* sequences clustered separately from Old World relapsing fever *Borrelia* spp. including *B. persica*, *B. hispanica*, *B. crocidurae*,* B. recurrentis*, and *B. duttonii*. The North American species including *B*. *hermsii, B. turicatae*, and *B. parkeri* clustered together and closer to the hard tick-borne RF species *B. miyamotoi*.

## Discussion

The current study represents the first description of *B. theileri* in Israel, providing new insights into this infection, with special emphasis on co-infection with *Babesia* and *Theileria* spp. within the bovine host population. To date, *B. persica* was the sole RF species reported in Israel with infection in humans, companion and wild animals, and ticks [7, 11–13, 33]. So far, only few reports of *B. theileri* describing its epidemiology and the clinical outcomes of its infection in animals have been published, and these originated from different locations in the world [18, 20]. Previous studies mostly describe highly variable molecular detection rates of *B. theileri* in *Rhipicephalus* spp. ticks, ranging from 0.3% in *R. annulatus* from Pakistan to 13.68% in *R. turanicus* tick pools from Iran. Lower detection rates of 0.54% and 2% were also reported in *R. geigyi* from Mali, and *R. microplus* from Brazil, respectively [21–24]. In contrast, information on the infection rates of *B. theileri* in cattle is sparse and includes a limited number of studies reporting 1.52% (92/132) in Holstein dairy cattle that displayed a drop in milk production and reduced food consumption in Brazil, 1.98% (2/101) in grazing beef cattle in Korea, and 4.1% (20/488) in beef cattle in Zambia [18–20]. Other studies reported *B. theileri* infection in cattle, with small study population sizes of up to 10 animals [34, 35].

In the current study, *B. theileri* infection was not associated with age or cattle production type (beef vs. dairy), indicating widespread exposure regardless of management practices. Although *B. theileri* infection was found to be significantly higher in bulls, this result should be interpreted cautiously because of the small number of males sampled. Furthermore, although rare cases of *B. theileri* infection in dairy cattle were documented [19], most reported data focuses on beef cattle infection. The current study demonstrates similar infection rates with *B. theileri* in beef and dairy cattle, suggesting considerable exposure of dairy cattle to tick infestation. Nevertheless, the proximity of beef cattle to dairy farms should be further evaluated as a risk factor for tick-borne pathogen infection. The geographical distribution of positive cattle covered diverse habitats, from urban environments to semi-arid and desert regions as is typical for southern Israel. This broad distribution of *B. theileri* highlights the adaptive and resilient nature of *Rhipicephalus* spp. ticks, as well as the wide distribution of cattle in Israel. Several *Rhipicephalus* spp. have been documented in Israel, including *R. annulatus*, *Rhipicephalus rutilus, R. turanicus*, *Rhipicephalus bursa, Rhipicephalus kohlsi, Rhipicephalus rossicus*, and *Rhipicephalus secundus* [36]. The potential vector capacity of these tick species requires further investigation to determine their role in *B. theileri* infection and for future consideration in surveillance and control programs.

Sequence identity of the *flaB* gene supports the classification of the detected isolates as *B. theileri*, showing complete identity with strains previously reported from ticks in Mali and Iran [21, 24], and from a sheep in Egypt (accession number PV551266.1). This high genetic similarity suggests low sequence variability and supports its reliability for species-level identification.

The detection of *B. theileri* in apparently healthy cattle, and in herds where cases of bovine piroplasmosis were detected, raises further questions regarding the clinical course of this disease in these infected cattle. An experimental study in a splenectomized calf reported only mild disease, limited to transient fever (40.2 °C; reference interval 38–39.2 °C) [37] and anemia (PCV 16.3%; reference interval 24–46%) [38] over a short period [6]. More recent studies describe variable outcomes, ranging from clinically normal cattle [35] to cases with depression, pale mucous membranes, mild fever, hemoglobinuria, and co-infection with other hemoparasites, occasionally resulting in death [39]. These observations suggested an involvement of *B. theileri* in the clinical presentation of these co-infected bovines [39].

Bovine piroplasmosis is prevalent in Israel and considered mainly as a beef cattle burden [40]. Furthermore, bovine piroplasmosis control programs in Israel rely on live attenuated vaccines developed for the immunization of beef, but not dairy cattle, to *B. bovis, B. bigemina* and *T. annulata* [41, 42]. In the current study, 6% of previously unvaccinated beef and approximately 3.5% dairy cattle were found PCR positive for *T. annulata* and *B. bigemina*. These findings indicate the presence of active piroplasmid infections in both production systems and suggest ongoing contact between cattle and tick vectors. Therefore, further research is warranted to investigate the current epidemiology of piroplasmid infections in dairy and beef cattle in Israel.

The limitations of this study included a small population of dairy and beef cattle tested, the small number of bulls and also a limited number of farms included in the survey. Furthermore, the *B. theileri* survey included a few farms where clinical evidence of piroplasmosis was diagnosed.

Further research would be helpful for the assessment of the clinical manifestations of *B. theileri* infection in cattle and the contribution of co-infection with other pathogens. Such studies may provide information on the need for targeted control strategies to minimize disease risk in the dairy and beef cattle populations. Furthermore, serological studies could be helpful for understanding the dynamics of this infection and co-infections and the degree of exposure to it in the cattle population.

## Conclusions

*Borrelia theileri* infection is present in cattle populations in Israel and appears to be underreported. The existence of this infection and piroplasmid co-infection in beef and dairy cattle populations highlight the widespread exposure to ticks and tick-borne pathogens in both cattle production systems, which requires the future implementation of control programs.

## Data Availability

All data generated or analyzed during this study are included in this published article. *B. theileri* sequences obtained in this study were deposited in GenBank as accession numbers PZ013538 ( *flaB*) and PZ013539 ( *glpQ*). *T. annulata* and *B. bigemina* sequences obtained in this study were deposited in GenBank as accession numbers PZ028038 and PZ028037, respectively.
